# Patient and therapist perspectives on impact, outcomes and change mechanisms in trauma-focused mentalization-based treatment: a qualitative interview study

**DOI:** 10.3389/fpsyt.2026.1808610

**Published:** 2026-06-02

**Authors:** Maaike L. Smits, Ilse M. Krah, Janine K. Driessen, Bram W. Kuppens, Arjen Noordhof

**Affiliations:** 1 De Viersprong, Viersprong Institute for Studies on Personality Disorders, Halsteren, Netherlands; 2 Department of Psychiatry, Section of Medical Psychology and Psychotherapy, Erasmus Medical Center (MC), Rotterdam, Netherlands; 3 Department of Clinical Psychology, University of Amsterdam, Amsterdam, Netherlands

**Keywords:** mentalisation-based treatment, trauma, complex trauma, qualitative, user perspectives, change mechanisms

## Abstract

**Introduction:**

Complex trauma is common in individuals with borderline personality disorder (BPD) and linked to persistent difficulties in emotion regulation, self-concept, and relationships. These co-occurring problems often lead to suboptimal outcomes in both trauma- and personality-focused treatments. Trauma-Focused Mentalization-Based Treatment (MBT-TF) is a structured group intervention developed to address complex trauma within a mentalizing framework. This study examined how participants and therapists experienced MBT-TF as an add-on to standard MBT for BPD, focusing on perceived impact, outcomes, and mechanisms of change.

**Methods:**

Six participants with BPD and a history of complex trauma and six therapists (two MBT-TF facilitators and four referring MBT therapists) were interviewed following completion of MBT-TF delivered alongside regular MBT. Semi-structured interviews were analyzed using reflexive thematic analysis. Three subsequent focus groups with therapist, participants and experts, refined and validated the emerging themes.

**Results:**

Two overarching domains carrying a total of seven themes were defined and validated through the focus groups. The first domain concerned the structured and relational conditions that enabled participants to tolerate the emotional intensity of trauma-focused work and reduce avoidance, grouping four themes: (1) facing emotional intensity together; (2) breaking avoidance in a safe, predictable, and consistent structure; (3) balancing trauma focus versus group dynamics; and (4) collaborative tailoring to individual needs. The second domain captured change processes in MBT-TF, comprising three themes: (5) varying experiences with symptomatic improvement; (6) changing perspectives on self and others through group experience; and (7) generalizing change beyond treatment. Change was mainly experienced as evolving through sharing, and perceiving oneself through the minds of similar others in a safe group context. This was associated with reduced shame, avoidance, and changes in self-other representations. Symptom change and generalization varied across individuals.

**Discussion:**

MBT-TF was experienced as tolerable and meaningful, with perceived change aligning with its core conceptual aims, and goals as formulated by participants. Findings suggest MBT-TF may serve as an alternative to or complement symptom-focused trauma treatments. Future research should explore stand-alone delivery, effectiveness, and change processes over time.

## Introduction

Trauma-Focused Mentalization-Based Treatment (MBT-TF) was developed as an adaptation of standard mentalization-based treatment (MBT, 1, 2), to address the enduring psychological and interpersonal impact of complex trauma within a mentalizing framework (3–5).

While MBT has demonstrated effectiveness for individuals with borderline personality disorder (BPD), both with and without histories of complex trauma (6), clinical experience underscores that for a substantial subgroup of patients trauma-related symptoms remain prominent despite engagement in standard MBT, undermining treatment success in traditional programs. This observation is supported by empirical evidence demonstrating that individuals with BPD and co-occurring trauma-related pathology - diagnostically classified as post-traumatic stress disorder (PTSD) or complex PTSD (CPTSD) - present with greater symptom severity, higher levels of dissociation and self-harm, and poorer treatment outcomes than those without trauma comorbidity (6–8). Epidemiological studies consistently indicate that a substantial proportion of individuals with BPD report histories of early or prolonged interpersonal trauma (9, 10), and that up to 30-50% meet diagnostic criteria for PTSD or CPTSD (11, 12).

Despite increasing recognition of this overlap, treatments for trauma and personality disorders have historically developed along parallel trajectories. Personality disorder (PD) treatments, such as MBT and dialectical behavior therapy, have demonstrated effectiveness in reducing general psychopathology, but may insufficiently address trauma-specific sequelae, including dissociation, trauma-related shame, and trauma-driven interpersonal avoidance (13). Conversely, trauma-focused therapies show reduced effectiveness in individuals with severe personality pathology (14, 15) and have demonstrated limited efficacy for complex PTSD (16, 17). Together, these findings indicate that neither trauma-focused nor personality-focused treatments alone adequately address the needs of patients with overlapping trauma-related and personality pathology, prompting calls for integrative and modular approaches that explicitly target both trauma-related processes and disturbances in self-relational functioning (18).

MBT-TF was explicitly designed to meet this need by embedding trauma processing within a mentalizing, relational framework (5), supported by a growing body of evidence linking impairments in mentalizing to the impact of complex trauma. Gorgellino et al. (19) in their systematic review showed a consistent negative correlation between childhood trauma and mentalization capacity, most profoundly in diagnostic groups of schizophrenia, depression and personality disorders, with neglect predicting the largest deficits in mentalizing. Moreover, studies have shown that mentalization mediates the effect of both attachment anxiety and avoidance on interpersonal distress (20). Mentalizing and epistemic trust have been shown to mediate the link between adversity and trauma-related symptoms (21) and improving mentalizing and epistemic trust can improve treatment outcomes for CPTSD, particularly the disturbances-in-self-organization domain (22). However, clinical experience indicates that merely strengthening general mentalizing capacity is often insufficient for individuals significantly affected by complex trauma, as improving general mentalizing capacity does not necessarily improve the ability to process traumatic memories or to manage their emotional and relational consequences. When left unaddressed, these disruptions can sustain cycles of shame, avoidance, and isolation and may limit the effectiveness of conventional PD treatments. While remaining grounded in the core principles of MBT, MBT-TF adds to standard MBT by introducing a more explicit trauma focus. Delivered in a group format, MBT-TF combines the structured, phase-based organization common in contemporary trauma treatments with an interpersonal context to break the pernicious impact of trauma-based beliefs about self, others and relationship (4, 23).

The intervention follows a clearly delineated sequence foregrounding trauma symptoms during assessment, followed by an explicit focus on the establishment of safety and trust, the collaborative exploration and mentalizing of trauma-related experiences, and a closing phase focused on grief, integration, and re-engagement with life. Rather than employing exposure-based techniques, MBT-TF aims to targets trauma-related disruptions of mentalizing, attachment, and epistemic trust that perpetuate cycles of shame, avoidance, and social withdrawal. Through this process, patients recalibrate distorted self–other representations, integrate fragmented self-states, and re-establish epistemic trust; the capacity to regard interpersonal information as reliable and meaningful. The mentalizing of trauma within relationships enables the transformation of unprocessed, isolating experiences into narratives that can be thought about, shared, and integrated, thereby restoring coherence and social connectedness (4).

Given the novelty and relational complexity of MBT-TF, qualitative methods are particularly well suited to explore how the intervention is experienced in practice beyond group-level efficacy, which obscures individual variation and the nuanced processes underlying change. By capturing participants’ and therapists’ lived experiences and subjective understandings of what is perceived as helpful or hindering, qualitative inquiry contributes essential experiential knowledge to psychotherapy research by elucidating subjective processes and meanings that remain inaccessible to quantitative outcome measures (24, 25).

The present study examines how MBT-TF was experienced by clients and therapists during its first clinical implementation as an add-on module within an MBT for BPD program for adults. The qualitative analysis explores the impact, process, and outcomes of MBT-TF, including participants perceptions of therapeutic impact, helpful and hindering processes, and mechanisms of change. In doing so, this qualitative investigation aims to provide an initial, practice-based understanding of how MBT-TF functions in routine clinical care and how it may contribute to recovery in individuals affected by complex trauma.

## Materials and methods

### Context, target population and participants

This study was conducted at De Viersprong, a Dutch mental health institution specialized in evidence-based treatment for personality disorders. The research took place at the Bergen op Zoom site, where MBT-TF was first implemented. operationalized as a six-month closed group add-on module to the standard MBT programs for adults with BPD. Patients were referred to MBT-TF by their MBT therapists when trauma-related pathology persisted despite ongoing MBT and was considered to hinder further therapeutic progress. Eligibility for regular MBT required participants to be aged 18 years or older, to have a primary diagnosis of BPD, and to experience significant distress and/or functional impairment in multiple social or occupational domains such as work, family life, friendships, or community functioning. In addition, patients typically presented with complex clinical profiles characterized by comorbidity, including other personality disorders, psychiatric disorders, physical conditions, and/or multiple concurrent psychosocial stressors. For participation in MBT-TF, additional criteria were: (a) a history of complex trauma; (2) willingness to discuss traumatic experiences within a group setting; (3) trauma-related difficulties in functioning entrenched at a representational level, involving maladaptive self-images, representations of others, and recurring destructive relational patterns linked to trauma-related experiences, for which standard MBT alone was considered insufficient; (4) agreement on an individualized crisis plan and the use of emotion regulation strategies.

In line with the MBT-TF manual, individuals engaged in therapy are referred to throughout this paper as *conversational partners* (CPs), reflecting the collaborative stance that underpins the therapeutic relationship in MBT-TF. The term *therapists* encompasses both *MBT-TF facilitators* and *referring therapists* from the primary MBT program. The term *participants* refers to all study participants, including CPs, MBT-TF facilitators, and referring therapists who took part in the interviews and focus groups.

A purposive sampling approach informed this retrospective study, including all CPs and therapists (four referring MBT therapists and two MBT-TF facilitators) involved in the first MBT-TF pilot. All eight CPs who entered the first MBT-TF group were invited to participate to the interviews; seven consented. One CP who discontinued treatment prematurely after two group session, was later interviewed to capture reasons for withdrawal, several months after completing treatment which may have influenced recall accuracy. For one CP, the interview recording failed due to technical issues and, although the interview informed the original internal evaluation, the absence of a verbatim transcript precluded inclusion in the thematic analysis. This CP did attend the focus group. This led to a total of six CP interviews to be included in the TA. Of therapist, in total six participated: two MBT-TF facilitators and all four referring MBT therapists. One therapist held dual roles within the study and was interviewed twice, with each interview focusing on the respective role (referring therapist and facilitator). **Table 1** provides an overview of relevant participant characteristics. Use of both groups allowed for triangulation between CP and clinician perspectives.

**Table 1:** Participant characteristics.

CP	Gender	Age	Treatment phase	Reasons for referral	
CP 1	M	51	follow-up care	Rigid negative core beliefs about self: being dirty, inferior	
CP 2	F	28	intensive treatment	Panic; intrusive bodily flash-backs; self-objectification	
CP 3	F	24	follow-up care	Rigid negative core beliefs about self and others; avoidance, interpersonal difficulties	
CP 4	F	41	follow-up care	Rigid negative core beliefs about self; avoidance of emotions and confrontations	
CP 5	F	23	intensive treatment	Flashbacks; rigid negative core beliefs about self and others; attachment avoidant; hypoarousal	
CP 6	F	26	intensive treatment	Rigid negative core beliefs; hypoarousal and dissociative symptoms	
Therapists	Gender	Age	Profession	MBT registration level	Work experience
Facilitator 1	F	45	social worker	MBT supervisor	21
Facilitator 2/RF 1	M	32	psychotherapist	MBT base level	9
RF 2	F	25	psychologist	MBT base level	4
RF 3	F	35	healthcare psychologist	MBT practitioner	13
RF 4	M	45	psychotherapist	MBT practitioner	10

RF, Referring Therapists; M, male; F, female; Work experience in years.

### Intervention

MBT-TF is a structured, phase-based group intervention designed to address trauma-related disruptions in mentalizing and epistemic trust, marked by shame, hypervigilance, and avoidance, driving social withdrawal and mental isolation that reinforce maladaptive self–other representations and perpetuate trauma re-enactment cycles (2, 4). In contrast to exposure-based trauma treatments, MBT-TF places primary emphasis not on the processing of traumatic memories per se, but on the restoration of social learning through new interpersonal experiences. MBT-TF is delivered in a group format and follows a clearly delineated, phase-based structure. It integrates principles from contemporary trauma treatment with the relational and epistemic focus of MBT. CPs are supported to reflect on traumatic experiences and their interpersonal consequences, in order to facilitate emotional processing without overwhelming affect or dissociative responding, using the group as a safe interpersonal context in which trauma-based beliefs about self and others can be examined, mentalized, and revised.

In the present study, MBT-TF was implemented as a six-month closed-group add-on module to standard outpatient MBT for BPD at De Viersprong. CPs from multiple ongoing programs were eligible for referral and together formed a newly constituted closed group, running parallel to their primary treatment trajectory. Eligible CPs were enrolled in various phases of the primary MBT programs, consisting of an intensive treatment phase entailing conjoint group and individual sessions, followed by a follow-up phase entailing individual psychotherapy only. Referral to the MBT-TF module could occur at different stages of the ongoing MBT trajectory - including the early, middle, or later phases of treatment - when MBT therapists judged trauma-related pathology persisted despite ongoing MBT and interfered with further therapeutic progress. Responsibility for overall treatment remained with the primary MBT team throughout the module. The MBT-TF add-on consisted of a preparatory individual phase of four individual sessions, among which one with significant others; followed by a structured group intervention with a total of 28 sessions during approximately seven months. The intervention adhered to an unpublished manual with respect to its phases and foci. The intervention’s rationale and structure are detailed by Smits et al. (4). The preparatory individual sessions took place between May 2023 and September 2023. The group phase of the MBT-TF module was conducted between 25 September 2023 and 22 April 2024. The initial preparatory phase and the final phase were condensed in length compared to MBT-TF as a stand-alone treatment, as CPs were already engaged in standard MBT, had previously completed a general ten-session psychoeducation group (MBT-I) prior to entering standard MBT, and were expected to continue in their primary treatment following completion of the MBT-TF module. Throughout the MBT-TF module, MBT-TF facilitators coordinated closely with the primary MBT team through regular consultation and input into regularly planned treatment evaluations to ensure continuity of care, support risk management, and promote the generalization of trauma-focused mentalizing gains.

*Preparatory individual phase (4 individual sessions)* - Prior to group participation, CPs completed four individual MBT-TF sessions, in at least one of which significant others were included to enhance support and continuity of care. These sessions aim to assess trauma history and current trauma-related symptoms, identifying triggers, dissociative symptoms and avoidant strategies, and mapping trauma-related reenactment cycles. Together with the facilitator, a shared case formulation was developed, including individualized treatment goals and the selection of trauma memories to be addressed during the group phase. Crisis planning and emotion regulation strategies were reviewed and refined.

*Group Phase 1: Stabilization and safety (6 group sessions).* The first group phase focused on establishing safety within the closed group setting. Sessions included psychoeducation on trauma and its effects, as well as the formulation of shared group values and agreements. Particular attention was paid to recognizing early signs of emotional overload, dissociation, and interpersonal rupture, and to strengthening affect regulation and mentalizing under stress. This phase culminated in a structured review session to assess group readiness to proceed to trauma processing.

*Group Phase 2: Trauma processing (18 group sessions).* In the trauma processing phase every CP is allocated two dedicated trauma-processing sessions. Processing sessions followed a fixed format, moving from guided individual recall and mentalizing of a selected traumatic memory to group-based mentalizing of its impact. Four review sessions were interspersed to monitor group safety, cohesion, and pacing.

*Group Phase 3: Integration, grief, and rehabilitation (4 group sessions).* The final phase focused on integrating trauma-related insights into a coherent self-narrative and addressing themes of loss, grief, and acceptance. Sessions emphasized the generalization of newly developed mentalizing capacities to relationships and daily life outside the group and explicitly prepared CPs for ending the group and continuing treatment within their primary MBT program.

### Design, procedure & data collection

The study employed a qualitative, exploratory design using a reflexive thematic analysis combining inductive coding with theory-based interpretive refinement (25), based on semi-structured interview data originally collected for internal evaluative purposes during the first pilot implementation of the MBT-TF add-on module at de Viersprong. Following recognition of the analytical value of the material, the existing interview data were repurposed for systematic qualitative analysis. The revised research plan was reviewed by the institutional research committe and deemed exempt from the Dutch Medical Research Involving Human Subjects Act (WMO) by ethical committee (METC Brabant). All CPs were subsequently re-contacted by interviewer J.D. and provided written informed consent for the use of their interview data for scientific purposes. All CPs were offered the opportunity to review the manuscript containing quotations to prevent identifiability. All CPs who consented to being approached for future research purposes were invited to participate in the focus groups.

The interview guide, based on a structured topic list and developed collaboratively with the MBT-TF development team, invited CPs and therapists to describe their perceptions of largely overlapping domains, including (a) reasons of referral to MBT-TF; (b) treatment goals; (c) the overall impact of MBT-TF, (d) helpful and hindering processes in the intervention. While content domains were similar for both groups, questions were phrased in a perspective-specific manner to reflect participants’ roles and capture experiential insights relevant to their involvement in MBT-TF (see supplemental materials - appendix I for interview guides). The interviews in this study reflect the experiences of CPs, referring therapist and the MBT-TF facilitators and were conducted between April 2024 and February 2025. CPs were interviewed 7–35 days after the end of the intervention (M=23 days), with the exception of one CP who discontinued treatment prematurely after two group session. She was retrospectively invited for an interview when the formal qualitative study was initiated, 505 days after her last MBT-TF session. Referring therapist were interviewed 31–80 days (M=85 days) and MBT-TF facilitators 245 days after ending the MBT-TF cycle. Interviews were performed via Microsoft Teams by a clinical psychologist (J.D.) experienced in MBT but not involved in the CPs treatment. She had no prior contact or therapeutic relationship with the CPs. Her familiarity with the program and treatment framework allowed her to discuss therapeutic topics easily while maintaining a neutral position, ensuring that the validity of the data was not compromised. Interviews lasted between 13 and 38 minutes (M=27), were audio-recorded, transcribed verbatim, and anonymized.

An inductive thematic analysis with theory-based interpretive refinement was conducted (25) to examine CPs and therapists’ experiences of MBT-TF. This approach was selected to generate rich, contextualized accounts of participants’ understandings of the processes and perceived effects of the intervention.

Methodological reporting adheres to the Standards for Reporting Qualitative Research (26) and Consolidated criteria for Reporting Qualitative research and principles of trustworthiness as defined by Lincoln and Guba (27) and applied following Nowell et al. (28). The study complies with the Declaration of Helsinki and national data-protection guidelines.

### Data analysis

Data were analyzed using a reflexive thematic analysis combining inductive coding with theory-based interpretive refinement, following the methodological steps described by Braun & Clarke (25). Data management and coding were supported by ATLAS.ti (version 25; 29). Data analysis was conducted by a multidisciplinary research team consisting of clinicians and researchers with backgrounds in psychology and psychotherapy research (I.K., B.K., I.M., J.B., M.S., A.N.). The analysis followed the six phases of thematic analysis, providing a systematic and transparent approach from initial coding to the development of final themes. In step 1, familiarization with the data was achieved by first transcribing all audio-recorded interviews into verbatim by the researchers themselves (lead coder I.K. and co-coders B.K., I.M. and J.B.) and subsequently read repeatedly to ensure immersion. To reduce subjectivity and increase rigor, in step 2, initial coding was carried out in multiple rounds. Four interviews were independently coded by all four coders to establish consistency and refine the codebook. In meetings following this, assumptions and choices were critically reflected upon, codes and interpretations were compared line by line, and differences in perspective were logged and resolved. Based on the refined codebook, lead coder I.K. coded the remaining eight interviews, and each co-coder independently coded three additional transcripts, so that all interviews were double-coded. This resulted into 580 initial codes that accurately reflected participants formulations. In step 3, the lead coder reviewed and merged the open codes into 92 axial codes representing recurrent meanings and processes, from which a preliminary thematic structure was developed of shared experiential patterns across participants. In step 4, this code tree and provisional themes were presented to the wider research team (including all co-coders, principal investigator M.S. and senior qualitative research consultant A.N.) for critical discussion. Divergent interpretations were logged and integrated through collaborative refinement of the thematic structure into a subsequent final thematic structure. In step 5 the prefinal themes were named, defined and outlined using representative quotes of participants, by I.K. in collaboration with the research team.

Across all analytic phases, data from CPs and therapists were examined within a single, integrated thematic analysis, rather than through separate group-specific analyses. Attention was explicitly directed to how emerging themes resonated across perspectives, as well as to differences in emphasis and interpretation, constituting an interpretive form of triangulation. As an additional step beyond the six analytic phases described by Braun and Clarke (25), three post-analysis focus groups were conducted to enhance the credibility and interpretive rigor of the findings. Two focus groups involved individuals directly engaged in the MBT-TF pilot and served as participant-informed validation of the thematic structure. These included (a) a therapist focus group, comprising both MBT-TF facilitators and two of the four referring MBT therapists (12 December 2025; 90 minutes), and (b) a CP focus group with experts-by-experience (five of the eight former MBT-TF CPs; 22 December 2025; 90 minutes). These sessions explored the resonance, clarity, and perceived completeness of the thematic framework. A third focus group functioned as an expert validation panel and consisted of nine international MBT-TF experts (A.B., D.B., P.L., S.T., L.N., J.d.V., J.D., T.N., E.R.) (15 December 2025; 90 minutes), providing conceptual and clinical reflections on the thematic framework from an external expert perspective.

In each session, the lead coder (I.K.) presented the data-driven thematic structure, after which participants were invited to share individual and collective reflections on the resonance, interpretability, and completeness of the themes, as well as potential blind spots and implications for interpretation and implementation. Discussions were moderated by the first author; field notes were taken, anonymized, and integrated through focused memoing. The *a priori* aim of these sessions was not to achieve consensus, but to enhance credibility and contextual fit by contrasting perspectives from clinical practice, lived experience, and conceptual expertise. Input from the focus groups informed final refinements to theme labels and interpretive emphasis, while leaving the overarching thematic structure intact.

### Rigor and reflexivity

Multiple strategies drawn from qualitative research standards (27, 28) were employed to enhance rigor, reflexivity, and transparency. Triangulation of data sources (CPs and therapists) and coders minimized idiosyncratic interpretation. Peer debriefing and critical-friend discussions within the research team fostered reflexivity regarding assumptions and interpretations. Credibility was further supported by the planned member-validation focus groups with (former) participants and the MBT-TF experts focus group to reflect on and refine the emerging thematic model. An audit trail was maintained throughout, detailing coding iterations and analytic decisions. Thick contextual description was used to facilitate assessment of transferability.

Reflexive practice was integral throughout the analytic process, including explicit attention to the potential influence of theoretical and clinical allegiance. The research team comprised clinicians and researchers with diverse backgrounds in psychology and psychotherapy, varying clinical seniority, and different disciplinary perspectives, and varying levels of pre-conceived knowledge of MBT-TF. Two team members had prior clinical experience with MBT-TF, while others had experience treating PD but were unfamiliar with MBT. The lead analyst (I.K.), a psychotherapist with formal training in qualitative methods and thematic analysis, had no prior MBT-TF experience, providing a “naïve” perspective on the model. A.N., a senior qualitative research consultant, contributed an external methodological perspective. Initial coding was conducted inductively by the lead analyst and remained closely grounded in participants’ language. MBT theory was intentionally set aside during early analytic phases and was introduced later as part of collaborative, iterative discussions aimed at theory-laden interpretive refinement rather than theory-driven coding, involving ongoing movement between data-driven coding, emerging interpretations, and theoretical and clinical perspectives. This allowed emerging themes to be considered in dialogue with MBT concepts without presupposing their relevance.

The team’s predominant clinical background enhanced contextual sensitivity to CPs’ language, therapeutic processes, and experiential nuances, while the inclusion of members without MBT-TF expertise and the external methodological perspective ensured critical distance and helped mitigate potential bias. Reflexive practices, including positionality statements, memo-writing, and critical team discussions, were used to surface assumptions, examine interpretations, and collaboratively refine emerging themes.

Analytic attention was directed both toward convergence and toward variation, ambiguity, and divergence within and across accounts. Divergent and disconfirming cases were thus treated as analytically informative rather than as outliers and used to refine, nuance, or delimit theme definitions.

## Results

The final thematic structure, developed through inductive analysis of CP and therapist interviews and subsequently refined through two validation focus groups, comprised seven overarching themes capturing how MBT-TF was experienced during its first clinical implementation. These themes represent shared perceptions of impact, therapeutic process, mechanisms of change and helping and hindering factors. In the following section, the themes are presented with illustrative quotations, followed by a summary of how the focus group reflections informed refinement and contextualization of the data-driven thematic structure.

### Final thematic structure

**Figure 1:**
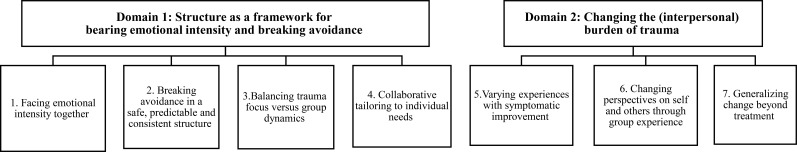
Thematic structure of MBT-TF-experiences. An overview of two overarching domains and seven themes, developed through thematic analysis of CP and therspist interviews and refined through three focus groups.

Through the process of reflexive TA seven themes were constructed that can be grouped into two broad domains (**Figure 1**). The first domain concerns the structured conditions that enabled participants to tolerate the emotional intensity of trauma-focused work and reduced avoidance. The second domain captures how MBT-TF shifted CPs trauma burden, especially in interpersonal domains rather than in specific PTSD symptomatology and the generalization of therapeutic gains into daily life. Below, we describe the two domains and their associated themes.

#### Domain 1: structure as a framework for bearing emotional intensity and breaking avoidance

This domain brings together themes that converged around the conditions required to engage in and sustain the emotionally demanding trauma-focused mentalizing work within MBT-TF. Four themes elaborate how structure, predictability, therapist emotional involvement and transparency, and individualized tailoring supported tolerability and counteracted trauma-related avoidance: (a) the emotional intensity of MBT-TF; (b) the role of structure in supporting tolerability; (c) the tension between trauma-focused work and group dynamics; and (d) the importance of individualized tailoring and timing of care.

#### Theme 1: facing emotional intensity together

Both CPs and facilitators consistently described participation in MBT-TF as emotionally intense, whereas referring MBT therapists articulated this less explicitly. For some CPs, engaging in MBT-TF felt like “*a big step*”, whereas others adopted a more neutral stance, entering the program “*open*[-minded]” or because it was proposed as part of their treatment by their therapist.

Sharing trauma was unanimously experienced as deeply challenging, particularly because difficulties with emotional expression and self-disclosure were often core reasons for entering treatment: “*It’s a trauma; it was actually a huge secret. I hadn’t spoken about it for 30 years. To then suddenly talk about it with others, that’s of course a huge leap.*” CPs also feared judgment: *“Of course, I was scared of what it would trigger in me, but also: how will people perceive me? Will they judge you?*” Listening to others’ trauma narratives was described as equally impactful, sometimes evoking memories, emotional reactions, or even flash-backs that “*briefly occupied*” them or “*were regularly brought home with them*”, alongside moments of reflection. The emotional strain of hearing others’ stories was experienced as substantial, yet usually manageable (“*I could empathize, but also keep in mind, that it was about someone else and leave it there.*”*)* and at the same time meaningful: *“It was intense, but afterwards I was glad I heard it.*” One CP remarked that the impact was more intense when others’ stories strongly related to their own experiences. Another CP *s*uggested that expressing feelings in the group helped regulate emotional intensity: “*I experienced no additional complaints from that. But I think that’s also because, when others told their story, we as a group were really able to express how this impacted us.*” The facilitators also experienced the module as emotionally intense and moving: “*The first sessions were very intense, as arousal was very high … things were said that touched me, as a person, and brought tears to my eyes.*” The other facilitator emphasized the valuable experience of connecting; “*I expected it to bother me a lot … But actually it wasn’t that bad. I genuinely thought it was really beautiful and special to do this with these people. It felt very together*”. As helpful in managing the emotional intensity, both facilitators mentioned the mutual and pleasant collaboration, their shared expertise and trust, the opportunity to jointly reflect on the group afterwards, and transparency about their own emotions within the group: “*We were all highly aroused; it was a difficult thing we were doing. And there was space to say that.*” Overall, despite the intensity, most CPs described the emotional load as tolerable. Nonetheless, adverse effects were reported by a minority: one CP ended MBT-TF prematurely due to cumulative stressors, while another described a worsening of physical complaints, noting that these “*really increased due to the stress*,” which led to missing the final session.

#### Theme 2: breaking avoidance in a safe, predictable and consistent structure

Structure and predictability were repeatedly described as crucial in managing emotional intensity. MBT-TF facilitators emphasized: “*The structure is very helpful. It’s clear: this is what we will do*.” Also, the majority of CPs referred to the consistent group and sessional structure, in a fixed order, and stable group composition increased safety: “*I really liked the structure. Every time there was an exercise and a go-round. It was always the same therapists, I really liked that too. You get to know each other and become familiar with how people respond. That really helps with sharing.*” The importance of predictability was also highlighted when discussing areas for improvement. One facilitator noted that although CPs were prepared in terms of which situation they would discuss, there was less preparatory focus on “*how* [this] *influences your interactional patterns and how you are going to talk about that.*” This, in their view, “*requires more preparation*,” particularly for the second trauma-processing session, in which CPs appeared to struggle with uncertainty about what was expected (“*what should I discuss now?*”; “*what do you expect from me?*”). The need for thorough preparation was echoed by several CPs, particularly those who experienced adverse effects or an increased physical burden. These CPs emphasized the importance of receiving “*more information about the treatment, what are the advantages and disadvantages, and the possible effects*.” Another CP highlighted how, over time, increased clarity about the sessional structure benefited later sessions: “*The first few sessions were less clear; as sessions progressed, it became more intense and you knew better what to expect*.” Overall, both CPs and therapists valued the structured trauma focus and the clear time and space allocated for processing trauma memories specially in comparison to regular MBT for BPD groups, the explicitly designated trauma-processing time was appreciated (“*two sessions just for me*”), contributing to a strong sense of safety and reducing avoidance: “*In the regular group, I could avoid easily. Here I had to. That really helped*”.

#### Theme 3: balancing trauma focus versus group dynamics

Several interviews highlighted the tension between maintaining a trauma-focused agenda and addressing frictions in group dynamics. At times, interpersonal tensions, group dynamics or challenging interaction patterns went unaddressed due to limited time and the felt need by the facilitators to move on to the trauma processing content. Also, one facilitator noted that ‘group suitability’ might need to be considered more explicitly considered at intake. In subsequent reflections, however, she framed the issue not as excluding CPs but as the need for better preparation for the predictable interpersonal re-enactments of trauma-related relational patterns that inevitably arise in a group-based trauma intervention, particularly salient in one case where such reenactments appeared to contribute to friction in the group: “*We didn’t explicitly discuss with her. that certain patterns would inevitably recur in the group … more preparation is needed*.” Experiences on how frictions in group dynamics were addressed were mixed: in some cases, expressing frustrations proved helpful as noted by a referring therapist; in others, CPs suppressed them due to limited space to voice such tensions, unlike in regular MBT group therapy. Several CPs and therapists described this as difficult, with one CP noting she had “*pushed away*” her frustrations. A facilitator described this as an ongoing balancing act: “*The puzzle was: your goal is trauma processing … but how much time do you spend on interpersonal work needed to keep the group safe?*” Some CPs experienced frustration when agreed-upon norms around attendance were not followed, when one group member dominated the sessions, or when opportunities for mutual recognition felt overlooked. One CP refers to the group norm of trauma sessions being strictly reserved for that person, diminishing opportunities for mutual recognition: “*Of course* e*veryone had their own session. Sharing part of your own story during these sessions didn’t always feel appropriate, even though when it happened, it was valuable.*” According to some referring therapists, experiences from the group were taken up in individual MBT sessions (“*During the individual sessions at the time, much of the focus was on what was happening in the group and how it affected her*”), illustrating how this group-based add-on module interacted with - and fed into - the individual component of the primary MBT treatment, and vice versa. This was also reflected by another referring therapist, who noted that “*the goals we had in the individual sessions also aligned with the TF goals, so in that respect, you really got a kind of coherent whole from it*”.

#### Theme 4: collaborative tailoring to individual needs

Therapists and CPS stressed that timing, suitability, and tolerability should be carefully and collaboratively evaluated when determining who should start MBT-TF and how it fits within their overall treatment trajectory. Indications for, and motivations to start with, MBT-TF alongside the regular MBT program were generally consistent. Most CPs entered the trauma-focused module following stagnation in previous treatments for long-standing trauma-related difficulties, often on the advice of their individual MBT therapist. One CP described making this decision in consultation with loved ones and a peer, stating: “*Of course I talked about it with my wife and with another group member … Then we decided together: well, you know what, we’re going to do it anyway, we’re going to take on that challenge*.” In most cases, referrals were experienced as appropriate by the TF facilitators; however, in some instances, doubts were expressed across those involved (referring therapists, facilitators, and CPs). Crisis-prone behavior was the most common source of hesitation: “*We both doubted because of the crisis risk … I worried the increased tension could be dangerous. [CP]*” and “*She became more and more destructive and then came that trauma group. I explicitly said to her: we’re going to have to think about this, because on the one hand I want to put you in that group, but on the other hand I can imagine that if we increase the arousal that much, it will be your end or something.*”[referring therapist]. Therapists emphasized in this context, the necessity of a crisis plan that participants could take responsibility for. Another source of doubt concerned trauma severity in the absence of a formal (C)PTSD diagnosis; “*Is it severe enough? Unlike the others I referred, she had never been diagnosed with PTSD.”* [referring therapist] *and “I did have doubts, because I was afraid that my story was not serious enough. The questionnaire showed that I did not have PTSD.*” Timing also played a key role in tolerability. When multiple stressors coincided, such as major life events, therapist changes, or transitions in primary treatment groups, MBT-TF was harder to manage. This convergence of stressors accounted for the only premature termination. One facilitator reflected that she “*did not think enough about timing*,” referring to a CP who “*eventually dropped out, because so many things happened in her life, so it really just wasn’t feasible*,” and emphasized that “*if we had predicted a little better what her resources were, not only in herself, but also in the broader context of her life at the moment … we might have predicted that this wasn’t the right moment for MBT-TF.*” Similarly, one CP described how the end of their regular MBT group coincided with the end of MBT-TF: “*I finished almost at the same time … so much was lost all of a sudden … I ended up sitting at home, having nothing else.*” In addition, CPs stressed the value of tailoring in-session exercises to their level of arousal. Finally, both CPs and therapists highlighted the importance of (previously acquired) mentalizing and emotion-regulation skills, with many noting that prior MBT experience helped them stay within their window of tolerance: “*Without the MBT I had before, I wouldn’t have managed. It helped me stay within my window.*” Another CP reflected: “*I had already benefited quite a lot from what I learned about myself in the regular MBT. Just the basics, the principles of mentalizing, opening up at all, connecting. The stepped approach now felt natural, building on what I learned in regular MBT.*”, and “*otherwise I would not have been able to do it*”. In line with this, the crisis-prone CP indicated that participating earlier, given her circumstances at the time, would have been “*something very dangerous.*”.

#### Domain 2: changing the (interpersonal) burden of trauma

This domain encompasses CPs accounts of the aims of MBT-TF and the extent to which these were realized through changes in how trauma was experienced, understood, and managed interpersonally. Three themes elaborate shifts in self–other representations, relational functioning, and emotion tolerance and regulation, as well as the emerging generalization of these changes beyond the treatment group.

#### Theme 5: varying experiences with symptomatic improvement

Most CPs did not report on MBT-TF as primarily targeting specific PTSD symptoms. In several interviews, such symptoms were not discussed at all, suggesting that they were not central to CPs expectations, lived experience, or perceived change. Instead, CPs described self-formulated treatment goals and observed changes related to self-image, interpersonal functioning, acceptance, and insight. Referring therapists and facilitators occasionally referred to classic PTSD symptoms such as intrusions or avoidance, yet similarly placed greater emphasis on broader trauma-related difficulties, particularly changes in self-image and interpersonal functioning. Only one CP articulated a classic PTSD-symptom-related goal of “*wanting to ‘go back’ less*” – which was almost completely achieved: “*I actually almost don’t have it anymore. I can’t remember when the last time I went back to that moment was*.” Another, despite not having articulated this as a goal, nonetheless reported clear PTSD symptom reduction: “*It feels very different, yes. I used to have a lot of nightmares, and they have become a lot less*”.

Across the sample, symptom change was variable: some CPs reported no clear symptom improvement, while others described fluctuations in symptom burden, including temporary worsening of emotional or physical complaints. One facilitator noted: “*I sense that for many people, no clear symptom reduction emerged; in some there has, in others there has not. I think this part may have lagged behind*.” Consistent with this, two CPs continued with exposure-based therapy or EMDR as part of their follow-up treatment to further address symptoms. One CP described “*a shift rather than an improvement in symptoms*”, with trauma-related tension and physical restlessness becoming more prominent: “*Yes, I do think that a difference has been made, but more that everything has just shifted a bit, instead of stating that it has improved. I notice that I find it more difficult to regulate my symptoms myself, also around other people*.” Overall, these findings indicate that perceived change was primarily reflected in domains such as emotion regulation and tolerance, self-image, and interpersonal functioning, and less consistently in direct reductions of classic PTSD symptoms; however, these observations are derived from open-ended interviews in which symptomatic change was not systematically or explicitly assessed.

#### Theme 6: changing perspectives on self and others through group experience

The fact that MBT-TF constitutes a group intervention was emphasized as a source of support and change. The majority described mutual recognition among group members as comforting and providing a sense of not being alone in their experiences: “*I discovered I’m not the only one. That was of most importance: it was comforting*.” The value of sharing experiences with others who had also been victimized was emphasized as meaningfully different from receiving support from people without lived trauma experience or from therapists. As one CP reflected, “*I think people who haven’t experienced that themselves might say, ‘Oh, that’s terrible for you, but they haven’t gone through it. They haven’t been through the same experience.*” A referring therapist further emphasized the unique impact of mutual recognition among peers, noting that “*support from someone with lived experience can sometimes feel more powerful than support from a therapist.*” Nonetheless, therapist involvement was seen as important for establishing safety and trust, with particular value placed on their transparency about emotional responses: “*Of course they have kept some distance as therapists, but it is human to be moved by someone’s story as well. And if you dare to show that in the group, I think that’s really powerful.*”.

Prior to MBT-TF, all CPs struggled - to varying degrees - with intimacy, forming relationships, and sharing personal experiences. A facilitator noted that “*being able to share more about their experiences, look back on it and feel connected…*” was a shared treatment goal among CPs, and this was achieved by everyone in the group: “*I think they all achieved that.*” Many described these new experiences of sharing and feeling connected as key drivers of change, particularly in contrast to earlier interpersonal experiences and expectations. Alongside these relational difficulties, almost all participants also described enduring trauma-related negative beliefs about themselves (“*I thought for a long time I deserved this or uh … I brought it on myself*”) and the majority of CPs also articulated their personal treatment goal in this reign: “*… improving my self-image was a very important goal.*” Sharing and discussing traumatic experiences challenged these entrenched negative views of the self and others, as mentioned in nearly all interviews: “*For me, I think the greatest shift came from hearing the stories of others that I could relate to. As a result, I looked at those people differently, but I was also very aware: oh, this is so very different from how I look at myself, although this is almost the same situation. That led to becoming more kind to myself.*” One CP reported that receiving and giving support strengthened feelings of self-esteem and confidence*: “I was also told by others that I had nice reactions and questions…”* A number of interviews showed that reactions from group members also had a recalibrating effect on feelings of guilt: “*What exactly those shifts are … I think the reactions of my group members were really sincere, which resulting in: Oh, okay. What happened was not okay and it happened to me, and I didn’t bring that on me. It’s not your fault. That really helped, so sincere people who are victims like me, who respond like: ‘Hey, how sorry for you.’ That really helped.*”.

As a result of these change processes, CPs described gradual shifts in how they perceived themselves and others, described as increased self-compassion, reduced self-criticism, and shifts in how they perceived others’ intentions: “*Some kind of a more compassionate voice began to emerge, that I am not doing everything wrong or am not so stupid, that I have the right to be here. This voice was really amplified by this TF group”*, and “*For a long time I thought of myself as dirty, a dirty person, a dirty individual, and now I can distinguish: I’m not dirty myself; what happened to me is dirty.*” Also the way they experience themselves as being perceived by others changed: “[I used to feel that] *they could see that in me, that it happened to me. I now experience this much less. I think I used to experience that I had a secret to keep, that people could see that I have a secret.*” In the majority of interviews, this increased self-understanding, mildness and self-compassion was put forwards, as articulated by a facilitator: “*the feeling of not being alone did a lot. But that is also really about self-image, about: I’m not crazy, I’m not bad, I didn’t do this to myself, I’m not guilty. So I think there was a lot of impact on self-image. And I also think that what we saw was that some people started to look differently at their context*”. CPs reported more “*space*” and “*understanding*” towards themselves and a decrease in self-criticism. Even a CP who experienced an increase in trauma-related distress described this increasing degree of self-compassion: “*At the same time, I think the trauma treatment has contributed to me being able to have a little more understanding for myself, even though it remains a constant struggle.*” One CP also mentions increased empathy and understanding for others: “*I started to think about people in a completely different way … sometimes people just do crazy things, but there may be something behind it. I started to think about that more.*”.

Both facilitators and referring therapists also observed changes in self- and other-representations, even when behavioral change lagged behind, although these observations were often expressed with some reservation (“*I’m not sure whether it produced the shift I had hoped for*”), emphasizing partial rather than complete change. As one referring therapist reflected: “*There has been a substantial shift in both self- and other-representations, but not unequivocally: he can still be distrustful of others, yet he no longer responds solely from that distrust and can act less impulsively*.” This tentative framing also underscored the perceived need for consolidation, aligning with theme 7 on variation in therapeutic gains and generalization.

#### Theme 7: generalizing change beyond treatment

For some CPs, the group experience and the newly developed perspectives generalized beyond the group into daily social and interpersonal functioning; such as asking for support or resuming social contact where participants were not able to before. CPs indicate that they have gradually opened up, to a greater or lesser extent, initially towards their group members, but some also towards their social environment. Several CPs aptly linked the internal shift to concrete behavior: “*I started asking for much more support, because I also gained much more confidence that I could get it”.* Others described sharing traumatic experiences more openly with significant others - “*I talked to my partner about it [ed: traumatic experience(s)]. He knew in general terms how and what, but now I’ve told more about how it went. That was also very new.*” - or breaking patterns of social avoidance: “*We recently went back to our church. I started to avoid that for a while, and now I go every Sunday again. And now I have contact with people again.*” This paralleled the observations of several referring therapists, of changes in attitude and relational engagement in their CPS following MBT-TF: “*I also notice it in our conversations. At a certain point he started to sit much more lively, to talk louder, literally to take up more space in his chair and to move more.*”.

At the same time, both facilitators and referring therapists emphasized that these shifts often required time to consolidate and did not always generalize immediately. One facilitator reflected: “*I don’t know if they can take it outside yet … I think that takes more time.*” Another was more reserved, noting that while sharing traumatic experiences had been achieved, broader changes in self-image or relational patterns were still emerging: “*I don’t know if it produced the shift I had hoped for … but I do think a seed has been planted that can grow*.” This cautious appraisal was mirrored by some CPs, for whom changes felt fragile or less extensive than anticipated: “*Of course, I had hoped it would bring me more*”.

Some CPs described that they had become more in touch with their emotions, recognized feelings better and were able to make connections that were previously difficult to access. One CP described this as accompanied by increased suffering: “*that was difficult to experience, because it then feels so big, while I actually had done my best for so long to keep it as small as possible*.” Therapists framed such increases in affective intensity not as setbacks, but as clinically meaningful openings. Referring to this CP, one MBT-TF facilitator understood the changes as “*an important first step”* and a referring therapist defined it as moving from “*pretend mode*” towards “*much more on an psychic equivalence mode of functioning”*, and a necessary precursor to further trauma-focused work for this CP: “*For her, it was really a very important first step to start feeling more space within herself to work with that trauma. That has made the treatment possible, and now this can become more of a topic in the follow-up treatment*.” Facilitators added that some CP’s (at least two) subsequently engaged in follow-up trauma-focused interventions (e.g., exposure or EMDR), describing MBT-TF as having created *“more openness and space”* in ongoing treatment to work with traumatic material more concretely - material that had previously been difficult to address due to re-experiencing or dissociation.

Overall, MBT-TF was widely experienced as *initiating* important processes rather than *completing* them, and the progress that was made seemed to vary between individuals, both in terms of direct benefits and degree to which CPs had been able to generalize their gains to life outside of treatment.

### Refinement through focus groups

Three focus groups were conducted to examine the resonance of the final thematic structure across perspectives (therapists, experts, and CPs). Importantly, these post-analysis focus groups were not intended as a consensus-building exercise, but as an opportunity to place the emerging thematic structure in dialogue with different forms of expertise, including clinical practice, lived experience, and conceptual knowledge. Overall, the thematic structure was widely recognized as coherent and clinically meaningful, with substantial convergence across groups in how themes were understood and appraised. Resonance was strongest in the therapist and CP focus groups, where themes were consistently experienced as recognizable and grounded in lived clinical experience. In contrast, the expert focus group - understandably given their greater distance from this specific intervention cycle and the fact that they did not contribute to the interview data on which the thematic analysis was based- yielded a more heterogeneous discussion, with relatively greater emphasis on broader conceptual, methodological, and implementation-related considerations. A more detailed account of focus group processes, theme-by-theme reflections, and of convergent and divergent perspectives within and between focus groups is provided in Supplemental Materials - appendix II. Here we provide an overarching synthesis.

Across all three focus groups, theme 6 (Changing perspectives on self and others through group experience) emerged as the most affectively resonant element of the thematic structure. CPs described these shifts - particularly changes in self-perception towards a more positive and compassionate view, evolving through the experience of seeing themselves through the eyes and minds of others - as among the most meaningful outcomes of MBT-TF. Therapists strongly recognized these representational shifts as central to the intended aims and working mechanisms of the intervention and were particularly affected by CPs accounts, given their uncertainty about whether such change had been sufficiently achieved in this first pilot cycle in light of their limited prior experience with MBT-TF. Experts similarly highlighted these shifts as a core mechanism, consistent with the theoretical foundation of MBT-TF, and as a potentially distinguishing feature compared to exposure-based trauma interventions, thereby lending support - in their perception - to the intervention framework and manual.

Second, a cross-cutting point across focus groups concerned the recurring recognition of theme 3, representing the clinical challenge of balancing a clear trauma focus with the need to actively address group dynamics, including relational tensions and trauma reenactment cycles. MBT-TF facilitators reflected on this as important “lessons learned” from this first pilot experience. CPs, in turn, emphasized the high value of the explicit trauma focus, while also noting that moments left unaddressed could undermine felt safety, and detailed several shared experiences illustrating this vulnerability. Experts likewise recognized this balance as a major therapeutic challenge across contexts, reinforcing the relevance of explicitly integrating relational mentalizing as an active mechanism rather than a competing process within trauma-focused group work.

A third salient point concerned the strong affective resonance of theme 7; capturing the variability in generalization of therapeutic gains beyond the group context, particularly within the CP focus group. For some CPs, MBT-TF appeared to reduce avoidance beyond group, and foster sustained openness to new social experiences, enabling continued growth through engagement in new interpersonal interaction. For others, however, reflecting on their experiences in MBT-TF highlighted a painful contrast between the intensity of connection experienced within the MBT-TF group and the current more limited support available outside treatment, re-evoking feelings of loneliness and prompting questions about how consolidation might be strengthened beyond therapy.

Beyond these strongly affectively resonant points, reflections across focus groups consistently affirmed the relevance of the remaining themes, including the centrality of safety, predictability, shared norms, and clear structure as preconditions for engagement, the importance of layered holding to tolerate the emotional intensity of trauma-focused work, and the emphasis on relational and self-representational change rather than symptom reduction, thereby further supporting the coherence and clinical meaningfulness of the thematic structure as a whole. Taken together, the three focus groups converged on a shared conclusion: MBT-TF appears to achieve early reductions in the burden of trauma primarily through restoring social connection and enabling participants to “see themselves through the minds of similar others,” within a structured holding context that supports tolerance of high emotional intensity. At the same time, there was strong convergence regarding the importance and fragility of generalization: relational gains achieved within the group were variably translated into everyday life, underscoring the need for deliberate scaffolding beyond the group context, including engagement of the broader relational environment and social network, as well as more integrated treatment evaluation and structured opportunities for consolidation, to help consolidate emerging changes and support generalization.

## Discussion

This qualitative study provides a systematic, in-depth, practice-based understanding of MBT-TF as it was experienced during its first clinical implementation as an add-on module within a standard outpatient MBT program for BPD. Thematic analysis of the interviews revealed how CPs and therapists experienced MBT-TF’s impact, process, and outcomes, yielding two domains comprising seven themes, refined through focus groups with therapists, experts, and CPs. The first domain concerned the structured, safe, and relational conditions that enabled CPs to tolerate the emotional intensity of trauma-focused work and reduce avoidance, encompassing four themes: (1) facing emotional intensity together; (2) breaking avoidance in a safe, predictable, and consistent structure; (3) balancing trauma focus versus group dynamics; and (4) collaborative tailoring to individual needs. The second domain captured how MBT-TF shifted CPs’ trauma burden, particularly in interpersonal domains rather than in specific PTSD symptomatology, and how therapeutic gains generalized into daily life, comprising three themes: (5) varying experiences with symptomatic improvement; (6) changing perspectives on self and others through group experience; and (7) generalizing change beyond treatment.

### A structured holding framework for tolerating emotional intensity and breaking avoidance

CPs and therapists consistently emphasized the structured format of MBT-TF as crucial for tolerating the emotional intensity of trauma-focused work. Clarity, predictability, and consistency were valued as central in promoting safety and engagement. The clear structure and explicit trauma focus helped both CPs and therapists in overcoming the inevitable avoidance that often arises in response to the impact of trauma. These findings closely align with those of a recent study by Bales and Kouijzer (30) which qualitatively explored the processes, impact and effects of a similar MBT-TF add-on module in a population of neurodiverse late adolescents, and likewise emphasized the importance of boundaries, safety and structure. Similarly, Chouliara et al. (31), in a qualitative study on a group-delivered interpersonal trauma intervention, highlighted that participants’ ability to open up, share, and express themselves in a group context was crucial for sustaining engagement in trauma-focused work. In all these studies, safety was positioned as a critical precondition for such openness, with predictability and psychoeducation identified as key elements. The clinical challenge of managing group processes alongside trauma content- captured in our third theme - also aligned with findings of Chouliara et al. (31), in which therapists emphasized the importance of balancing attention to how trauma is processed within versus what content is addressed.

Our findings resonate with established models of trauma treatment, which emphasize safety, stabilization, and psychoeducation as prerequisites for trauma processing (17, 23, 32). Within MBT-TF, however, these elements appeared to function not merely as preparatory stages, but as active mechanisms of change. Predictability, psychoeducation, and the phase-bases structured approach were considered essential in supporting mentalizing under high affective arousal, thereby enabling sustained engagement with the trauma work.

### Changing the interpersonal burden of trauma through shifts in self-other representations

Most salient to the domain of change was the shared understanding across triangulated focus groups - captured in theme six - that MBT-TF’s early reductions in the interpersonal burden of trauma were primarily achieved through restoring social connection and enabling CPs to see themselves through the eyes and minds of similar others within a holding group context. This process appears to support early shifts in self-other representations (e.g., reduced self-blame, increased self-compassion, revised expectations of others). This mechanism appeared to be central and affectively salient for both CPs and therapists and was conceptually endorsed by experts, consistent with the theoretical assumption that change within this mentalizing trauma intervention is driven by the (re)establishment of epistemic trust, social connection and referencing, rather than by exposure-based fear activation or habituation processes. This aligns with previous findings Chouliara et al. (31) which showed that being with others alike reduced feelings of aloneness and supported continued participation -a process also identified as a key driver of change in qualitative research on mentalization-based interventions non-specific to trauma (33). This may reflect a specific mechanism of change: mentalizing the self through being mentalized by ‘someone like me.’ This mechanism relates to the idea that social openness and epistemic trust are more readily activated in interactions with others perceived as similar, as shared experience fosters a sense of being seen, which has been proposed as a prerequisite for social learning (34, 35). In line with this, Bales and Kouijzer (30) found that shared neurocognitive profiles fostered mutual understanding among neurodiverse late adolescents.

Recent network analyses of CPTSD further contextualize these findings. Karatzias et al. (36) demonstrated that negative self-concept constitutes the most central symptom cluster among individuals with histories of childhood poly-traumatization, relative to symptoms clusters of avoidance, threat perception, or re-experiencing. In this context, the finding that MBT-TF primarily facilitates change through shifts in self–other representations is particularly encouraging. In our study, participants repeatedly emphasized the centrality of an altered self-view, typically moving towards a more compassionate and less self-blaming stance. Rather than targeting symptoms directly, MBT-TF thus appears to engage underlying mechanisms by addressing trauma-induced negative views of self and, subsequently, altering expectations of others’ intentions, thereby which may support new forms of interpersonal engagement.

Across interviews, CPs and therapists consistently described both the goals and perceived effects of MBT-TF as centered more on changes in self-other representations than on symptom relief. Change was experienced as increased emotional tolerance, reduced shame, and improved interpersonal functioning, supporting further therapeutic work or ongoing growth beyond treatment. In contrast, symptom change varied: while some CPs reported symptom reduction, others noted shifts without clear symptom improvement, or even temporary increases in distress. At least two CPs engaged in follow-up trauma treatments such as EMDR or exposure therapy. This relative emphasis on relational over symptomatic change, captured in themes 5 and 6, aligns with prior qualitative studies stressing the importance of attending to the pervasive ways in which trauma affects individuals’ lives and sense of self, rather than focusing narrowly on symptoms (31). This is consistent with Bales & Kouijzer (30) who found variable symptom change, whereas changes in self-image, self-worth and relationships and social interaction were more consistently reported and formed distinct thematic themes. This also suggests that evaluating MBT-TF - or trauma treatment more broadly - through symptom-based outcomes alone may miss key therapeutic gains, paralleling literature stating that patients tend to value therapeutic outcomes beyond symptom reduction, emphasizing deeper self-understanding, increased agency, and improved social functioning (37). Instead, a broader framework towards recovery may be needed, capturing experiences of meaning, worth, and belonging as important aims (38).

Notably, the observation emerging from this study that MBT-TF may primarily influence the relational impact of trauma, facilitating shifts in self-other representations - whereas other trauma treatments may more directly target symptom clusters such as flashbacks or nightmares - has potential implications for dissemination strategies and clinical decision-making. Broader literature emphasizes the value of matching trauma interventions to the primary domains of distress or dysfunction (36). It also highlights the potential clinical utility of a more differentiated or sequential approach, raising the question of whether MBT-TF and other trauma focused interventions should be matched to specific symptom profiles, or - particularly in complex cases where trauma is deeply embedded in both representational processes and persistent symptom clusters - understood as serving complementary roles within an integrated treatment trajectory.

### Understanding MBT-TF mechanisms in practice and clinical implications

Despite prevailing concerns among clinicians that trauma-focused interventions might be too emotionally demanding for individuals with complex trauma and personality pathology and that group based formats may be particularly risky in such populations, MBT-TF was overall experienced as tolerable, even in the face of high emotional intensity. Moreover, the group format was highly valued by both CPs and therapists, and no indications of retraumatization or escalating crises were reported within this pilot sample. Tolerability was consistently linked to well-implemented factors of structure, predictability, and consistency.

Referring therapists and facilitators elaborated (in focus group, see supplemental material - appendix II) on how feasibility and safety were also supported by *layered holding structures* that provided scaffolding for the emotional demand of the trauma-focused work. Facilitators embedded within the group carried the most immediate and affectively intense impact alongside the CPs, while referring therapists positioned alongside the group - in this add-on module configuration - offered a stabilizing presence and helped CPs tolerate heightened arousal from a slightly greater distance. Notably, the notion of how emotional intense MBT-TF is to CPs was more explicitly articulated by the facilitators than the referring therapist, revealing an experiential gap between being embedded within the intervention versus observing it from a supportive distance. The concept of layered holding structures may highlight the need to more explicitly involve CPs’ significant others in the MBT-TF process and to prepare them, including through targeted psychoeducation, for the emotional demands of treatment. In the present implementation, such involvement was largely confined to a single session during the assessment phase, and in some instances, the post-MBT-TF treatment evaluation. More explicit relational scaffolding, particularly when MBT-TF is delivered as a standalone intervention without a parallel MBT infrastructure, by mobilizing CPs’ social networks may be essential for supporting emotional tolerance. Moreover, assessing CPs’ relational context and available external support may also inform decisions on timing and feasibility and promote the generalization of therapeutic gains into everyday life.

Although predictability was consistently regarded as essential factor in managing emotional intensity (theme 2), both CPs and therapists indicated that this element was not yet optimally implemented and could be strengthened in future cycles. In the initial add-on implementation, psychoeducation and preparation were relatively condensed due to CPs’ prior MBT experience, among which general psycho-educational preparatory groups (MBT-I). As a result, preparation for the trauma-focused work specifically appeared as relatively limited. This first cycle thus revealed a need for more trauma-specific preparatory work, even among those familiar with MBT, particularly with regard to the anticipated emotional and bodily impact of trauma processing. Relatedly, missed opportunities in fostering predictability became apparent in relation to the emergence of trauma-related reenactment cycles. In hindsight, both CPs and facilitators reflected that interpersonal tensions that temporarily affected safety in group could be understood as predictable reenactments of trauma-related relational patterns. This prompted therapists to reflect on the clinical challenge - and importance - of integrating trauma focus with group dynamics, rather than treating them as competing demands. Both facilitators and CPs indicated that clearer and more explicit preparation, anticipating on the likelihood of such dynamics would have been helpful. From an implementation perspective, this points to the value of (1) identifying potential trauma reenactment cycles from the assessment phase onward, not only at the individual level but also collaboratively within the group, and (2) increasing in-session transparency around therapeutic decisions when balancing trauma focus with attention to group dynamics. Importantly, these suggestions do not indicate deviations from the MBT-TF model. The MBT-TF manual explicitly delineates the therapeutic use of reenactment processes and relational mentalizing of trauma. Rather, findings highlight a learning curve in early implementation and reinforce the importance of fully consolidating manual-based principles, especially during early cycles.

Readiness for emotionally demanding trauma work emerged as a recurring clinical consideration, as captured in theme 4 and substantiated through focus group discussions with both CPs and therapists. This was seen to depend not only on individual factors such as emotion regulation capacity, crisis vulnerability, and daily stability, but also on the availability of external relational scaffolding during and beyond treatment. Prior MBT experience was widely reported by CPs as highly facilitative, and for some even essential, for developing the basic capacities needed to tolerate MBT-TF. Therapists largely concurred, observing that the group progressed without crises, potentially due to skills gained in the parallel MBT trajectory. However, views differed on whether such experience should be considered a strict prerequisite. The current implementation and qualitative study design did not allow for direct comparison between CPs with and without prior MBT exposure. Experts further noted that these perceptions may reflect the add-on configuration of the current implementation, in which CPs could directly compare MBT-TF to their ongoing MBT, shaping their engagement and perceived readiness in ways that may not generalize to stand-alone delivery.

Decisions regarding eligibility and timing may be best approached in a collaborative and context-sensitive manner, balancing safety with flexibility without unnecessary restriction or dilution of the group-based format. While flexibility in the use of individual sessions to support stabilization or consolidation may be clinically appropriate in some cases, the affectively resonant mechanisms identified in this study - such as the recalibration of self-other representations through shared experiences and mentalizing interactions with ‘others-like-me’- are uniquely tied to the group format. Preserving the integrity of MBT-TF as a primarily group-based intervention thus seems essential to its core therapeutic potential. At the same time, future implementation and research efforts may further explore stand-alone delivery, including the role of individual support and other context-dependent adaptations.

Finally, challenges of generalization beyond the group context were also salient (theme 7). Although shifts in self-other representations and reductions in mental and social avoidance were widely recognized, their translation into everyday life appeared variable and highly context dependent. This is consistent with the mentalization-based framework, which conceptualizes enduring change as depending not only on in-session learning, but also on broader social communication processes through which new understandings and relational experiences are consolidated in everyday life (35). CPs’ accounts suggest that the transition from an intense relational environment in therapy to everyday contexts characterized by limited support can be particularly challenging and may contribute to posttreatment loneliness for some individuals. In the present addon implementation, the ending phase of MBT-TF was relatively condensed, partly based on the assumption that continued consolidation would be supported within the broader MBT program. However, for some CPs, the end of MBT-TF coincided with the end of their overall treatment trajectory, potentially amplifying experiences of discontinuity and loneliness. From a clinical perspective, this underscores the importance of a sufficiently elaborated ending phase and suggests that structured follow-up -such as periodic group based booster sessions - may support consolidation and generalization, particularly when MBT-TF is delivered as a standalone intervention.-treatment loneliness for some individuals. In the present add-on implementation, the ending phase of MBT-TF was relatively condensed, partly based on the assumption that continued consolidation would be supported within the broader MBT program. However, for some CPs, the end of MBT-TF coincided with the end of their overall treatment trajectory, potentially amplifying experiences of discontinuity and loneliness. From a clinical perspective, this underscores the importance of a sufficiently elaborated ending phase and suggests that structured follow-up -such as periodic group-based booster sessions - may support consolidation and generalization, particularly when MBT-TF is delivered as a stand-alone intervention.

### Methodological considerations and limitations

Several methodological considerations should be taken into account when interpreting the findings. First, this study combined and jointly analyzed data from CPs and therapists. While this approach allowed for an integrated understanding of MBT-TF processes, it is well established that CPs’ and therapists’ perspectives may differ substantially (39–41). Analyzing these perspectives separately might have yielded additional or divergent themes. The decision to integrate perspectives was guided by the study’s aim to understand MBT-TF from a shared understanding, co-constructed across roles, along with pragmatic consideration regarding the limited number of participants in each group. This integrative approach was further supported by the use of validation focus groups, which explored the resonance, credibility, and meaningfulness of the findings among both former CPs and therapists, thereby mitigating the risk of assumed convergence between these groups. Second, the retrospective nature of the interviews and the fact that data collection initially served internal evaluation purposes may have influenced the findings. Interviews were not originally formatted to fit the means of systematic qualitative research purposes. The retrospective nature of interviewing introduces recall bias, potentially limiting for instance insight into within-treatment fluctuations. Variability in interview timing may have reduced consistency across accounts. For one participant specifically, who had dropped out of MBT-TF, a substantial longer time lag occurred between treatment termination and the interview. Although this delay had clear practical reasons, the extended interval may have heightened potential recall bias effects, with accounts potentially being influenced by subsequent life events or further therapy or by placing greater emphasis on integrated meanings rather than immediate treatment experiences. Nevertheless, the convergence of narratives across participant groups and the iterative, reflexive analytic procedure strengthens the credibility and ecological validity of the findings. Third, the study reflects the inherent challenges of evaluating an evolving intervention during its pilot phase. Since data collection, MBT-TF has undergone further refinement and standardization. Moreover, the facilitators were still in the early stages of developing experience with this specific intervention format, which may have shaped both delivery and reflections on the process as well as participant selection. Specifically, as MBT-TF was introduced as a newly developed intervention, it is possible that this first group of CPs held a relatively specific position, characterized by heightened interest, curiosity, expectations or openness toward innovation. Such early-adopter effects may have contributed to more favorable appraisals, while perspectives of more reluctant or less engaged participants may be underrepresented. This limits transferability to other implementation context, where referral pathways, expectations, and familiarity with the model may differ. This is partly mitigated by the inclusion of the CP who terminated treatment prematurely. Moreover, from a broader clinical perspective, tailoring interventions to patients’ wishes, needs, and readiness - and supporting realistic expectations and hope at treatment entry - is widely conceived as general factor of treatment success and constitutes a primary aim of all MBT-based interventions. Future cycles of delivery may specifically benefit from the involvement of experts-by-experience in preparatory stages, to support a realistic appraisal of what participation in MBT TF may entail, enabling patients to enter treatment with sufficient readiness and openness. Fourth, despite the use of multiple safeguards to mitigate potential theoretical and clinical allegiance effects - including inductive coding, team reflexivity, and the involvement of analysts with varying familiarity with MBT-TF - the involvement of members of the research team in the development and dissemination of the intervention means that some degree of allegiant influence on interpretation remains possible and should be considered when interpreting the findings. Fifth, the treatment manual underlying the intervention under study has not yet been formally published, which limits full transparency and reproducibility. As MBT-TF is a developing intervention, manual publication is pending the completion of ongoing effectiveness and feasibility studies to which the present study contributes. Finally, as the data are based on a small, relatively homogeneous sample drawn from a single early implementation cycle, the extent to which these findings transfer beyond the current context remains an open question. The findings should therefore be understood as capturing an early developmental stage of the intervention, providing insight into the mechanisms and implementation processes as inferred, experienced and reported within the current context, rather than providing definitive evidence regarding intervention effects, and may inform ongoing model development, therapist training, and future cycles of delivery.

### Future directions and collaborative knowledge development

Future research should examine MBT-TF across a broader range of clinical contexts and investigate both its impact and mechanisms of change using more rigorous confirmatory designs with systematic assessment of symptom change and others outcomes, and in-depth qualitative designs, such as realist evaluation, in more diverse samples. In particular, it will be essential to study how trauma-focused mentalizing unfolds across treatment phases and how it relates over time to changes in symptom burden, relational functioning, and epistemic trust, as well as to clarify how contextual factors shape engagement, safety, and outcomes. A key question concerns the extent to which MBT-TF, as a primarily group-based intervention, can be effective as a stand-alone treatment versus requiring embedding within a broader treatment infrastructure. The group-based foundation of MBT-TF is driven by mentalizing theory on the need for social re-connection (42–44) but also has direct implications for treatment accessibility and scalability. If MBT-TF proves to be effective as a predominantly group-based standalone intervention, this would strengthen opportunities for upscaling and broader availability compared to individual or combined formats. Future research should therefore explicitly examine the relative contributions of different intervention components (e.g., group-based versus individual scaffolding elements), using both quantitative designs and qualitative approaches to capture how these components operate in practice across different implementation contexts. Finally, these findings highlight the importance of positioning MBT-TF within the broader landscape of trauma interventions. While MBT-TF was developed to address a clinical gap for individuals with complex trauma and associated difficulties in personality functioning, current findings suggest that its primary impact may lie in facilitating shifts in self–other representations and interpersonal functioning, whereas changes in PTSD symptoms appeared less explicitly targeted and more variable, based on qualitative, experiential accounts rather than systematically assessed outcomes. These insights call for further investigation into how MBT-TF’s mechanisms compare to or complement those of symptom-focused interventions, particularly in terms of matching treatment components to distinct dimensions of complex trauma presentations. This underscores the need to move beyond comparative effectiveness questions framed solely in terms of superiority, toward research that compares trauma interventions in terms of target pathology, target populations, mechanisms and processes of change. Such work may support shared decision-making regarding differential indication and clarify when, particularly in more complex presentations, different trauma-focused approaches may need to be sequenced, combined, or integrated as complementary components that support personalized care and comprehensive recovery.

Notably, both CPs and therapists expressed a strong motivation to remain engaged in dialogue about the study’s findings and their implications. This shared reflective engagement suggests opportunities for participatory and co-productive research approaches, in which therapists, (former) CPs, and researchers collaboratively reflect on outcomes, refine intervention components, and shape future implementation. Such ongoing dialogue may itself embody core MBT-TF principles, reinforcing epistemic trust and supporting the iterative development of relational-based trauma treatments.

## Data Availability

The datasets presented in this article are not readily available because the datasets generated for this study consist of sensitive qualitative interview data. Requests to access the datasets should be directed to maaike.smits@deviersprong.nl.
