# Supplementary material for: Patient and therapist perspectives on impact, outcomes and change mechanisms in trauma-focused mentalization-based treatment: a qualitative interview study
Source: Front Psychiatry. 2026 Jun 2;17:1808610. doi: 10.3389/fpsyt.2026.1808610 (PMC13269095; doi:10.3389/fpsyt.2026.1808610)
Supplement: Supplementary file 1 [file DataSheet1.pdf]

frontiers

Supplementary Material – Appendix I - Interview guides

Patient and therapist perspectives on MBT-TF

Therapist interview

(For the referring therapists of the primary MBT for BPD program)

	**Referral/Indication criteria for treatment**
1a	What was the reason for indication/referral to the MBT-TF module?
1b	What consideration(s) have been made in the referral?
1c	How do you look back on the referral and what did you learn from it?
	**Goals and changes**
2a	What treatment goal did the participant work on in MBT TF?
2b	To what extent has the goal been achieved?<br>*Not at all – slightly - almost completely - completely*
3	What changes did you notice in the participant after following the MBT TF module?
4	What was not accomplished?
5	Did the MBT-TF module impact the primary MBT for BPD treatment (group/sociotherapy/individual therapy), if so how?
6	What would you recommend to improve MBT-TF in future cycles?
	**Perception of MBT-TF and collaboration**
7	What is your perspective on the MBT-TF module?

Appendix I – Interview guides

8	Was there an added value of following MBT-TF in addition to the regular MBT program in the participant's process?
9	How did you experience collaboration with the MBT-TF therapists in the different phases (from indication/referral to completion)?
10	What would you have wanted differently?
	**Concluding**
11	Do you have any other remarks, suggestions or questions regarding the evaluation of MBT-TF?

` **Participant version**

	**Referral/Indication criteria for treatment**
1	What was the reason for indication/referral to the add-on MBT TF module?
2	Did you or your therapists (of the primary MBT for BPD program) have any doubts with regards to starting MBT-TF?
	**Goals and changes**
2a	What treatment goals have you been working on in MBT-TF?
2b	To what extent have you achieved your goal?<br>*Not at all – a little – almost completely – completely*
3	Can you describe in your own words what you have achieved in relation to this goal?
4	What was not accomplished?
5	What other effects have you noticed, both within your primary MBT for BPD treatment and/or in daily life?
6	What would you recommend to improve the intervention?

	**Experience with MBT-TF**
9	What is your perspective on the MBT-TF module?
10	Was there any added value for you compared to the regular MBT for BPD program?
11	What would you have wanted differently?
	**Concluding**
14	Do you have any further comments, suggestions or questions regarding the evaluation of the MBT-TF module?

MBT-TF facilitators

	**Referral/Indication criteria for treatment**<br>(question about general observed trends and individual examples)
1.a	What was the reason for indication/referral to the add-on MBT-TF module?
1.b	What consideration(s) were made in the indication/referral?
1.c	How do you now reflect back on the indication process, and what are lessons learned?
	**Goals and changes**
2a	What treatment goals did the participants work on in MBT-TF?
2b	To what extent did participants achieved their goals?<br>*Not at all – slightly - almost completely - completely*
3	What changes did you observe in the participants after following the MBT-TF module?
4	What was not accomplished?
5	Did the MBT TF module have an impact on the participant's regular MBT for BPD treatment (group therapy/sociotherapy/individual therapy)? If so, how?

Appendix I – Interview guides

6	What would you recommend for future implementation to improve MBT-TF?
	**Experience with MBT-TF and collaboration**<br><br>(question about general observed trends and individual examples)
7	What is your perspective on the MBT-TF module?
8	Was there an added value of following MBT-TF in addition to the primary MBT program in the participant's process?
9	How did you experience collaborating with the fellow therapists in the different phases (from referral/indication to completion)?<br><br>• With the co-MBT-TF-facilitator<br>• With the referring therapists/teams from the primary MBT program
10	How did you experience facilitating this trauma-focused intervention? (e.g., impact of hearing emotionally intense or traumatic experiences)
11	Were you able to maintain your own mentalizing stance? What helped or hindered you in doing so?
12	What would you have wanted differently?
	**Concluding**
13	Do you have any further comments, suggestions or questions regarding the evaluation of the MBT-TF module?
