# Supplementary material for: Patient and therapist perspectives on impact, outcomes and change mechanisms in trauma-focused mentalization-based treatment: a qualitative interview study
Source: Front Psychiatry. 2026 Jun 2;17:1808610. doi: 10.3389/fpsyt.2026.1808610 (PMC13269095; doi:10.3389/fpsyt.2026.1808610)
Supplement: Supplementary file 2 [file DataSheet2.pdf]

Supplementary Material - Appendix II - Detailed account of focus groups

Patient and therapist perspectives on MBT-TF

Three focus groups were conducted to enhance the credibility and interpretive rigor of the findings by examining the resonance of the final thematic structure across perspectives. Importantly, these post-analysis focus groups were not intended as a consensus-building exercise, but as an opportunity to place the emerging thematic structure in dialogue with different forms of expertise, including clinical practice, lived experience, and conceptual knowledge.

Note that, in line with the MBT-TF manual, individuals engaged in therapy are referred to throughout this appendix as *conversational partners* (CPs). The term *therapists* encompasses both *MBT-TF facilitators* and *referring therapists* from the primary MBT program. The term *participants* refers to all study participants, including CPs, MBT-TF facilitators, and referring therapists who took part in the interviews and focus groups.

Two focus groups involved individuals directly engaged in the MBT-TF pilot and served as participant-informed validation of the thematic structure. These included (a) a therapist focus group, comprising both MBT-TF facilitators and two of the four referring MBT therapists (12 December 2025; 90 minutes), and (b) a CP focus group with experts-by-experience (five of the eight former MBT-TF CPs; 22 December 2025; 90 minutes). These sessions explored the resonance, clarity, and perceived completeness of the thematic framework. A third focus group functioned as an expert validation panel and consisted of nine international MBT-TF experts (A.B., D.B., P.L., S.T., L.N., J.d.V., J.D., T.N., E.R.) (15 December 2025; 90 minutes), providing conceptual and clinical reflections on the thematic framework from an external expert perspective.

Here, we present a summary of reflections from the therapist, expert, and CPs focus groups in chronological order, with specific attention for both convergent and divergent perspectives within and across focus groups. Disagreement between focus group members and/or the research team was explicitly welcomed and treated as analytically informative rather than resolved into consensus. Input from the focus groups informed refinements at the level of theme labels and interpretive emphasis, while the overarching thematic structure remained intact. Overall, feedback from the focus groups largely confirmed the coherence and relevance of the final thematic structure and the delineation of the respective themes.

Therapist focus group

The focus group with the two MBT-facilitators and two referring therapists largely confirmed the relevance of the identified themes. Therapists explicitly recognized the importance of safety, safeguarded by predictability and consistency as a fundamental precondition for engagement and as key facilitating factor in the intervention (validating theme 1 and 2). Safety was discussed as being shaped by both intervention-related and contextual factors. Intervention-related factors emphasized by the therapists included the importance of shared group norms and adhering to them (including clarity about attendance expectations), early sessions psycho-education to enhance predictability regarding

what CPs might expect in terms of the intervention's impact. Facilitators also recognized the importance of a clear structure, to which they articulated additionally that discontinuities in roles (e.g., assessments conducted by a therapist who was not subsequently involved in trauma-narrative coaching during the individuals' processing session) may undermine felt security, highlighting the value of deliberate role continuity among the facilitators. The difficulty of balancing trauma work with managing group dynamics or potential conflict in group was recognized (validating theme 3) and therapists reflected upon the fact that mentalizing the relational impact of trauma, and explicitly identifying trauma re-enactment cycles might have aided in difficulties in group relationships, which stresses the importance of good preparation in this respect, and having this explicitly return in the group.

Safety was also supported by the sense of *holding* created within and around the intervention. This included the therapists' emphasis on CPs being familiar with fellow group members, facilitators' emotional involvement, and the broader contextual support available outside the sessions. Such containment, both relational and structural, was described as crucial for managing the emotional intensity of trauma-focused work, for both CPs and therapists, thereby reinforcing the significance of Theme 1 and Theme 4. (thereby validating Theme 1 and Theme 4). Referring therapists and facilitators elaborated on their shared experience of a "*layered holding structure*" or "*holding circles*" that provided scaffolding for the emotional demands of the trauma-focused work: facilitators embedded within the group carried the most immediate and affectively intense impact alongside the CPs, while referring therapists positioned alongside the group - in this add-on module configuration - offered a stabilizing presence and helped to tolerate heightened arousal from a slightly greater distance. They further reflected that such layered holding structures may be particularly critical when MBT-TF is implemented as a stand-alone intervention, in the absence of a parallel (standard) MBT treatment infrastructure. In such contexts, CPs' social networks may need to (be facilitated to be able to) take on a more explicit supportive role, underscoring the importance of psychoeducation for significant others about the anticipated emotional intensity and potential impact of the intervention. Therapists noted that the emotional intensity was more directly felt by therapists embedded within the group than by those positioned outside it, revealing an experience gap between being inside versus outside the intervention. This underscored the importance of clearly communicating the emotional burden to CPs' significant others.

Therapists noted that, despite the high emotional intensity of the work, the group unfolded without crises or severe dysregulation, which raised the question of whether a prior MBT trajectory - and the associated strengthening of emotion regulation and mentalizing capacities - may have functioned as an enabling condition. While therapists indicated that such capacities are likely to be highly facilitative, they did not reach consensus on whether prior MBT experience is strictly necessary, as the current implementation, and qualitative evaluation thereof, did not allow for that comparison. Finally, therapists noted that when implemented as an add-on module, coordination between the MBT-TF facilitators and primary MBT team was important for treatment coherence highlighting the need for clear agreements, role clarity, and consistency, elements therapists emphasized as standard within mentalizing interventions and as part of general adherence to the model.

Last, but perhaps most importantly, therapists in the focus group strongly resonated, at an affective level, with CPs' descriptions of shifts in self-perception and relational understanding (i.e., shifts in self–other representations) (validating Theme 6), which they recognized as conceptually and clinically

central to the aims of MBT-TF. At the same time, therapists reflected a degree of uncertainty regarding the extent to which these intended representational changes had been achieved, related to their felt challenges in this first pilot cycle and their own limited prior experience with MBT-TF. The facilitators described having drawn important lessons learned that, in their view, aligned with implications as presented by the thematic structure. These included the need to (a) prepare more explicitly for the expected effects and burden of trauma work (for both CPs and their significant others), (b) more clearly anticipate and address trauma-related reenactment cycles, thereby strengthening the mentalizing of trauma's relational impact (a core aim of MBT-TF) while simultaneously enabling group dynamics to be used as an active therapeutic mechanism rather than experienced as competing with trauma processing. In relation to generalization (validating Theme 7 and converging with CPs' emphasis on generalization as a salient and resonant theme, see below in 'CPs focus group') therapists additionally reflected that a stronger and more explicit focus on relational mentalizing, both within the processing sessions and particularly during the ending phase of MBT-TF, might have further supported the transfer of therapeutic change beyond the group context; they also viewed this as partly related to their limited experience with this intervention format.

Expert focus group

The expert focus group included both developers of the MBT-TF model and clinicians with experience facilitating MBT-TF groups in other contexts, who had not been involved in this specific intervention cycle. Understandably, given their greater distance from the current implementation and the fact that they had contributed to the interview data on which the thematic analysis was based, this focus group yielded a more heterogeneous and fragmented discussion. This appeared partly process-related and may reflect the variation in clinical contexts (e.g., MBT-TF as an add-on module versus a standalone format), and differing relationships to the intervention (e.g., as developers or external clinicians). In contrast to the CP and therapist focus groups, which were marked by shared and resonating experiences, the expert discussion placed relatively greater emphasis on broader conceptual, methodological, and implementation-related considerations. Accordingly, the expert focus group primarily contributed to clarifying broader conceptual and contextual considerations relevant to the intervention, and to informing future implementation and research efforts, rather than validating themes through experiential resonance. In addition, the pilot status of the intervention - being a first-time experience for all involved - emerged as an important context for interpreting findings. Several elements described by experts as "missing" appeared to reflect limited exposure to the format or early-stage implementation learning, rather than true omissions from the intended model. Experts also noted that, because MBT-TF was implemented as an add-on module, CPs were uniquely positioned to compare it to MBT-as-usual, which may have influenced their expectations and experiences differently than in standalone delivery. Finally, experts provided methodological input on theme phrasing, emphasizing the importance of staying close to participants' own wording. This feedback was integrated into the final theme labelling; specifically for theme 6 by changing the prefinal title version "Seeing myself through others: group as catalyst for self-other recalibration" into the final theme title "Changing perspectives on self and others through group experience".

Overall, experts broadly recognized the thematic structure, with particular emphasis on shifts in self–other experience as the most salient and unifying finding. These representational recalibrations were identified as fundamental to the original aims of MBT-TF and described as highly recognizable, not only by the model developers but also by the clinical experts across other populations and clinical settings. Recalibration emerged as the most consistently shared mechanism of change, with experts noting that CPs' accounts of "seeing themselves through the eyes of others" closely align with MBT-

TF's theoretical foundations and may distinguish its mechanism of action from that of i.e. exposure-focused trauma treatments. In this respect, the interviews were experienced by the experts as affirming for the clinical manual, as participants described meaningful shifts in self-perception and reduced self-criticism without reliance on exposure-based techniques.

Structure was reflected upon as a facilitating factor, helping to reduce avoidance in both CPs and therapists during this trauma-focused work. Experts recognized balancing trauma work with group dynamics (theme 4) as a major clinical challenge across contexts, without a straightforward solution, requiring careful therapeutic navigation. They supported the notion that facilitators' interventions could more explicitly reflect how group dynamics are intentionally used to support mentalizing the relational impact of trauma - an emphasis already embedded in the manual, which underscores the importance of initiating this process, using the concept of trauma re-enactment cycles, from the assessment phase onward into the group.

Another dominant focus of expert discussion concerned the perceived necessity of prior MBT for benefiting from MBT-TF. Experts questioned whether the consistent accounts from CPs and therapists - suggesting that prior MBT was at least highly facilitative, and possibly a prerequisite for tolerating and benefitting from MBT-TF - reflect an intrinsic requirement of the intervention itself or rather a contextual effect of its add-on implementation. While some experts emphasized the potential value of parallel supportive interventions to enable sufficient therapeutic impact, others framed the perceived "need" for individual sessions (in addition to group work) as potentially reflecting clinician anxiety or a protective tendency in the face of perceived risk. Within this discussion, experts noted that referring therapists appeared overall more confident and positive about what MBT-TF had generated than facilitators themselves. This discrepancy was interpreted as highlighting the potential value of intervision, supervision, and careful monitoring of overly cautious facilitation styles or inadvertent underestimation of progress by facilitators. The immediate emotional involvement of facilitators may instigate a strongly felt responsibility for the emotional safety of the group which could lead facilitators to underestimate therapeutic change. This interpretation converged with findings from the therapist focus group regarding the importance of layered holding structures in which therapists positioned outside the processing sessions remain actively involved.

Notably, in contrast to the other focus groups, Theme 5 elicited relatively more response among experts. The drafted label ("Relational rather than symptom goals: mixed experiences of change beyond symptom reduction") was seen as insufficiently capturing the breadth of participants' accounts, particularly regarding the variability in symptom change. This led to changing the theme title into: "Varying experiences with symptomatic improvement" in the final thematic structure description. One expert expressed surprise at how little PTSD as a diagnosis was mentioned in the interviews, especially by CPs (i.e. whether diagnosis was formally established, or whether symptoms had remitted). Based on cross-national clinical experience, this expert observed that diagnostic framing and PTSD symptom remission tended to be a more explicit focus in other groups than appeared to be the case here.

Experts also identified several aspects that appeared relatively underrepresented in the current interviews, despite being considered salient from their conceptual and clinical perspectives. These included (a) the embodied dimensions of mentalizing and the potential value of explicitly targeting the bodily impact of trauma within trauma-focused mentalizing work, (b) the explicit articulation and evaluation of group norms and values (while not prominently labelled in the thematic structure, this

was strongly emphasized in the other two focus groups), and (c) the role of shame as a potentially key mechanism of change. Regarding shame, experts discussed the clinical relevance of initially unlabelled affective states that may become accessible through naming and symbolization, drawing on both clinical experience and componential theories of emotion. One expert described that explicit work on shame in other trauma-focused groups facilitated relief and integration, whereas others emphasized that the relative absence of shame as an explicit theme in CPs' accounts may itself be meaningful, highlighting the interplay between subjective experience and symbolic labelling of shame for therapeutic change. These reflections also encouraged further conceptual differentiation between mechanisms specific to trauma-focused mentalizing and those reflecting more general MBT processes (i.e. MBT in general focusses on affect differentiation, labelling and naming of affects and emotions, and this may represent less of a unique MBT-TF focus which may explain why this was underrepresented as key mechanism in the interviews).

Experts concluded that the current results underscore both the promise of MBT-TF and its theoretical and clinical framework, as well as the limitations of the current data for evaluating the intervention more generic across context (i.e. as standalone treatment). This was integrated into the interpretation of our results in the Discussion section.

CPs focus group

The emotional intensity of MBT-TF (theme 1) resonated strongly with CPs and converged with therapists' reflections. CPs articulated that prior emotion regulation skills and mentalizing capacity had functioned as important prerequisites for engaging in MBT-TF; all CPs mentioned how these capacities facilitated participation, and some questioned whether they would have been able to take part without this foundation. Conversely, they also reflected on how MBT-TF fed back into their primary treatment trajectory, further strengthening their overall mentalizing capacity (i.e. their ability to "see behind one's own behaviour"). Theme 2 was also broadly recognised: CPs reflected on MBT-TF's clear structure and group cohesion - facilitated by explicit group norms and stable group membership - as key ingredients in breaking long-standing avoidance. The explicit articulation of group norms was described by some as "somewhat school-like", and CPs reflected that more collaboration in this process might have been helpful, while still recognising the importance it had. At the same time, they noted that adherence to certain agreements sometimes felt too non-committal. Deviations from shared norms - such as inconsistent attendance despite prior group agreements - were described as reducing feelings of safety, underscoring the importance of dedicating sufficient time to establishing shared norms in early sessions and subsequently upholding them reliably. Relatedly, CPs recognized - consistent with theme 3 - an ambivalence regarding the group's clear and somewhat exclusive trauma focus. The explicit trauma focus was highly valued, experienced as legitimising and containing, and recognised as an important driver of change. At the same time, CPs emphasised the need to also address relational events and tensions occurring within the group itself, as leaving these unspoken - paradoxically precisely because of the explicit trauma focus - in some instances contributed to feelings of unsafety. Some CPs described defensive reactions from other group members as emotionally arousing or destabilising, and reflected on how such reactions - while understandable to them as trauma-related protective strategies of this individual - could nevertheless trigger their own patterns in turn. They conjectured that it might have been helpful if the group had been more prepared to recognise each other's triggers, including those that might arise within the group context. Notably, these reflections converged with therapists' perspectives (see above) that, in clinical terms, more explicit identification of trauma reenactment cycles - and bringing these dynamics into the group process - had at times been limited, and that addressing them more explicitly may warrant greater focus, both to anticipate

unhelpful group dynamics and to (thereby) strengthen relational mentalizing regarding the interpersonal impact of trauma. Although being in a group was described as difficult - and at times experienced as unsafe - CPs consistently reflected on the importance of the group format. They spoke about the value of being with others who felt "alike", sharing vulnerability, and developing shared engagement, which supported the courage to open up about trauma. Across the discussion, CPs reflected on how being in a group helped break avoidance and gaining new social experiences that were non-confirmatory of trauma-related negative expectations about self and reflected on shifts in their views of themselves and others (validating theme 6).

CPs linked this to (variability in) the experiences of change beyond the group (validating theme 7). Reflections repeatedly circled back to generalization as the most dominant and affectively resonant theme in this focus group. Differences in how gains translated into daily life emerged both verbally and affectively. Some described continued change after MBT-TF, resulting from increased awareness, greater self-compassion, and a growing readiness to break avoidance and engage in new relational experiences, grounded in more positive self–other views. In contrast, one CP described heightened loneliness after the end of MBT-TF, which coincided with the conclusion of her broader treatment. This emotionally re-evoked sense of isolation was echoed during the focus group, as this CP recalled the strong connection within the group compared to their more limited current support. The gap between relational intensity in therapy and reduced connectedness outside made the transition to everyday life particularly challenging, which was recognized by others. CPs did not clearly specify what might have supported the generalization of change more strongly, and tended to frame social support as an important contextual factor outside the intervention itself. Although early MBT-TF sessions included discussion about the need for a "safety net," some CPs now reflected that they had underestimated just how critical social support would be - not only for enduring the intervention, but also for sustaining gains, reconnecting socially, and continuing change after treatment. Others noted that this aspect felt beyond their own - or their therapist's- span of control. Despite the challenges that were broadly recognized in generalizing therapeutic gains, experiences of success were also articulated. Two CPs reflected that they had become more inclined to continue challenging social avoidance and 'expose' themselves to others, even though this remained difficult and influenced by contextual stressors or life circumstances. One CP explicitly described that, through MBT-TF, they had come to experience how important and helpful it was to keep moving against avoidance, even when this required ongoing effort. CPs also reflected on how being in a process of change inevitably affects one's environment: significant personal change may create relational challenges, as others may not know how to respond or adapt. As one CP put it, "*When planting a seed, growth requires others for the seed to take root, yet others do not always respond in ways that support this process*."

Notably, the focus group discussion contained relatively little spontaneous elaboration in response to theme 5, which concerned the variability in symptom change and relational versus symptom-oriented goals. When the theme was explicitly probed, CPs reflected on the relational gains they experienced - or the absence thereof. In response to follow-up questions, CPs recognised that their goals had largely been formulated in relational terms, and the discussion shifted back towards reflections aligned with theme 7.

In addition to what emerged from the thematic structure, CPs reflected on the importance of shared treatment evaluation moments. They noted that evaluation opportunities, which were accommodated within their primary MBT trajectory, largely limited MBT-TF specific evaluation to written feedback

from the MBT-TF facilitators, which did not fully meet their need for shared, in-person reflection on the process and for collaborative adjustment when needed. This highlights the clinical relevance of integrating structured process evaluation moments, offering opportunity for joint reflection and recalibration of treatment processes. This aligns with the sense of shared responsibility and "doing it together" that CPs experienced as a hallmark of the intervention and highly valued. In this context, therapists' openness about the intervention being a first pilot experience was also explicitly appraised as authentic and collaborative rather than undermining credibility.

Finally, CPs explicitly expressed their interest to jointly reflecting on the current findings and their interpretation together with facilitators, expressing curiosity about their perspectives and a wish to contribute to the future development of the intervention.
